# Semantic VPS for Smartphone Localization in Challenging Urban Environments

**DOI:** 10.3390/s21186137

**Published:** 2021-09-13

**Authors:** Max Jwo Lem Lee, Li-Ta Hsu, Hoi-Fung Ng

**Affiliations:** 1 Department of Aeronautical and Aviation Engineering, The Hong Kong Polytechnic University, 11 Yuk Choi Rd, Hung Hom, Hong Kong; max.jl.lee@connect.polyu.hk (M.J.L.L.); ivannhf.ng@connect.polyu.hk (H.-F.N.); 2 Research Institute for Sustainable Urban Development, The Hong Kong Polytechnic University, 11 Yuk Choi Rd, Hung Hom, Hong Kong

**Keywords:** localization, navigation, smartphone, VPS, urban canyons, pedestrian, GNSS, BIM, 3D building models

## Abstract

Accurate smartphone-based outdoor localization systems in deep urban canyons are increasingly needed for various IoT applications. As smart cities have developed, building information modeling (BIM) has become widely available. This article, for the first time, presents a semantic Visual Positioning System (VPS) for accurate and robust position estimation in urban canyons where the global navigation satellite system (GNSS) tends to fail. In the offline stage, a material segmented BIM is used to generate segmented images. In the online stage, an image is taken with a smartphone camera that provides textual information about the surrounding environment. The approach utilizes computer vision algorithms to segment between the different types of material class identified in the smartphone image. A semantic VPS method is then used to match the segmented generated images with the segmented smartphone image. Each generated image contains position information in terms of latitude, longitude, altitude, yaw, pitch, and roll. The candidate with the maximum likelihood is regarded as the precise position of the user. The positioning result achieved an accuracy of 2.0 m among high-rise buildings on a street, 5.5 m in a dense foliage environment, and 15.7 m in an alleyway. This represents an improvement in positioning of 45% compared to the current state-of-the-art method. The estimation of yaw achieved accuracy of 2.3°, an eight-fold improvement compared to the smartphone IMU.

## 1. Introduction

Urban localization is essential to the development of numerous IoT applications, such as the digital management of navigation, augmented reality, and commercial related services [1], and is an indispensable part of daily life due to its widespread application [2]. For indoor areas, Wi-Fi based localization has become extremely popular and many researchers are focused on this area [3,4,5]. However, the use of Wi-Fi in urban areas is still highly challenging, and positioning is limited to an accuracy of tens of meters, even in strong signal conditions [6]. As indicated in [7], the calibration of Wi-Fi fingerprinting databases and the density of Wi-Fi beacons in urban areas pose a large number of challenges. As a result, Wi-Fi is mostly suitable for indoor positioning. In the context of outdoor pedestrian localization, the application of the global navigation satellite system (GNSS) is key to providing accurate positioning and timing services in open field environments. Unfortunately, significant improvement is needed in the positioning performance of GNSS in urban areas due to signal blockages and reflections caused by tall buildings and dense foliage [8]. In these environments, most signals are non-line-of-sight (NLOS), which can severely degrade the localization accuracy [9]. Hence, they cause large estimation errors if they are either treated as line-of-sight (LOS) or not used properly [10]. Therefore, efforts have been devoted to developing accurate urban positioning systems in recent years. A review of state-of-the-art localization was published in 2018 [11]. Each of these technologies has its own advantages and limitations. However, some of these solutions face other challenges, such as mobility, accuracy, cost, and portability. A pedestrian self-localization system should be sufficiently accurate and efficient to provide positioning information [12]. Currently available personal smartphones are equipped with various embedded sensors, such as a gyroscope, accelerometer, and vision sensors. These sensors can be used for urban localization, and also satisfy the requirements of being inexpensive, easy to deploy, and user friendly.

With the increase in the development of smart cities, 3D city models have been developed rapidly and become widely available [13]. An idea known as GNSS shadow matching was proposed to improve urban positioning [14]. It first classifies the received satellite visibility by the received signal strength and then scans the predicted satellite visibility in the vicinity of the ground truth position. The position is then estimated by matching the satellite visibilities. Another method is the ray-tracing-based 3D Mapping Aided (3DMA) GNSS algorithms that cooperate with the pseudo-range has been proposed [15]. The integration of shadow matching and range-based 3DMA GNSS is proposed in [16]. The performance of this approach in multipath mitigation and NLOS exclusion depends on the accuracy of the 3D building models [17]. In recent years, interest has increased in inferring positions using 3DMA and vision-integrated methods. The motivation is that these are complementary methods, which in combination can provide rich scenery information. This is largely because high-performance modern smartphones provide cameras, and computing platform for storage, data processing, and fusion, which can be easily exploited. The general idea behind most of these approaches is to find the closest image to a given query picture in a database of position-tagged images (three-dimensional position and three-dimensional rotation, adding up to six degrees of freedom [DOF]).

Research has demonstrated that it is possible to obtain precise positioning by matching between a camera image and a database of images. One popular approach uses sky-pointing fisheye camera equipment to detect obstacles and buildings in the local environment [18]. When used in conjunction with image processing algorithms, this approach allows the matching of the building boundary skyplot (skymask) to obtain a position and heading.

To date, several studies have examined the use of smartphone images to estimate the position of the user. Google’s recently developed feature-based visual positioning system (VPS) identifies edges within the smartphone image and matches these with edges captured from pre-surveyed images in their map database [19]. The position-tagged edges are stored in a searchable index and are updated over time by the users. Another area of study focuses on semantic information, such as identifying static location-tagged objects (doors, tables, etc.) in smartphone images for indoor positioning [20]; however, reference objects are often limited in outdoor environments. Thus, other researchers have studied the use of skyline or building boundaries to match with smartphone images [21,22,23,24]. This provides a mean positional error of 4.5 m and rotational error of 2–5° in feature-rich environments [21].

Although both methods are suitable in urban areas where GNSS signals are often blocked by high-rise buildings, the former requires features extracted from pre-surveyed images for precise localization, suffers from image quality dependency, and requires frequent updates using the cloud-sourced data supplied by users. By comparison, the latter suffers from obscured or non-distinctive skylines, which are prominent in highly urbanized areas where dynamic objects dominate the environment. Thus, detection based solely on the edges and the skyline may not be sufficient for practical use and precise positioning. From the perspective of pedestrian navigation, in addition to the identification of features and the skyline, humans also locate themselves based on visual landmarks that consist of different semantic information, for which each semantic has a material of its own. These high-level semantics are a new source of positioning information that does not require additional sensors, and many modern smartphones are already equipped with high-performance processors that can identify these semantics. These models are steadily improving in accuracy, and currently obtain accuracy of about 85% in city landscapes [25].

Therefore, inspired by existing methods, our proposed solution applies the semantic VPS by utilizing different types of materials that are widely seen and continuously distributed in urban scenes. The proposed method offers several major advantages over the existing methods.

- First, we take advantage of building materials as visual aids for precise self-localization, overcoming inaccuracies due to a non-distinctive or obscured skyline, which are common in urban environments.
- Second, the semantic VPS uses building information modeling (BIM), which is widely available in smart cities, due to its existing use in construction, thus eliminating the need for pre-surveyed images. Hence, it is highly scalable and low cost.
- Third, unlike storing feature data as 3D point clouds in a searchable index, the semantics of materials are stored as the properties of the objects in the BIM, enabling simple and accurate updates to be undertaken.
- Finally, the proposed method identifies and considers dynamic objects in its scoring system, which have usually been neglected in previous studies.

Thus, this study comprises interdisciplinary research that integrates the knowledge of BIM, geodesy, image processing, and navigation. We believe this interdisciplinary research demonstrates an excellent solution to provide seamless positioning for many future IoT applications.

The remainder of this paper is organized as follows. Section 2 explains the overview of the proposed semantic VPS approach. Section 3 describes the candidate image generation, material identification, and image matching in detail. Section 4 describes the experimentation process and the improvement of the proposed algorithm is verified with existing advanced positioning methods. Section 5 presents the concluding remarks and future work.

## 2. Overview of the Proposed Method

**Figure 1:**
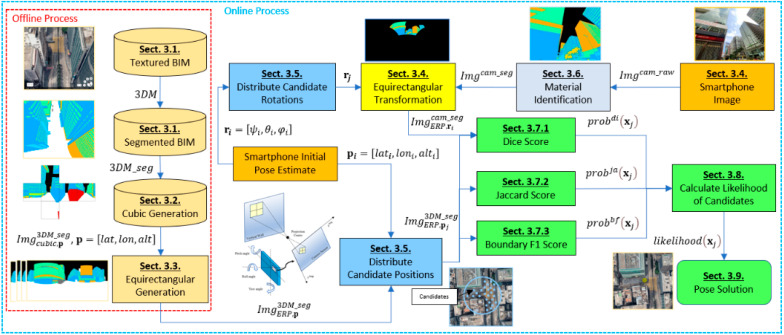
Flowchart of the proposed semantic VPS based on segmented smartphone images and segmented generated images.

An overview of the proposed semantic VPS method is shown in Figure 1. The method is divided into two main stages: an offline process and an online process.

In the offline process, the building models are segmented into different colors based on the material, which ensures a perfect representation of the materials in the BIM (Section 3.1). The segmented city model is used to generate cubic projections at each position (Section 3.2), which are then converted into equirectangular projection images (Section 3.3) for later comparison. By storing the images in an offline database within the smartphone, we can derive a memory-effective representation of accurate reference images suitable for smartphone-based data storage.

Based on the generated images, we propose a semantic VPS method for smartphone-based urban localization. In the online process, the user captures an image with their smartphone (Section 3.4), with the initial position estimated by the smartphone GNSS receiver and IMU sensors. Then, candidates (hypothesized positions) are spread across a search grid based on the initial position (Section 3.5). The smartphone image is then segmented based on the identified types of materials (Section 3.6). The segmented smartphone image is transformed into the equirectangular projection image (Section 3.4) to be matched with the candidate images using multiple metrics to calculate the similarity scores (Section 3.7). The scores of each method are combined to calculate the likelihood of each candidate (Section 3.8). The chosen position is determined by the candidate with the maximum likelihood among all the candidates (Section 3.9). The details of the proposed method are described in the following section.

## 3. Proposed Method in Detail

### 3.1. Textured and Segmented BIM

The city model used in this research was provided by the Surveying and Mapping Office, Lands Department, Hong Kong [26]. It consists of only buildings and infrastructure; foliage and dynamic objects are not represented in the models. Each building model consists of a level of detail (LOD) 1–3, stored in Autodesk Revit Format. In BIM, each object in the model has its own corresponding object name.

Because each object in the building model already contains a corresponding name, a color can be assigned for the material the name represents, which can then be used to efficiently simulate a segmented BIM, as shown in Figure 1, and allows fast scalability of a BIM map. In this research, we used six classes to test the feasibility of the proposed method. Each class has its own respective RGB color: Sky (black), Concrete (blue), Glass (green), Metal (orange), Foliage (yellow), Others (light blue).

The city model uses the 3D Cartesian meter coordinate system on a plane to determine the positioning coordinates. Therefore, it was necessary to convert the measured GNSS positioning information in (latitude and longitude) to the 3D Cartesian coordinates. Thus, we transformed between the WGS84 Geographic coordinates and Hong Kong 1980 Grid coordinates using the equations described by the Surveying and Mapping Office, Lands Department, Hong Kong [27].

### 3.2. Cubic Projection Generation

Each projection and its respective coordinate systems require careful clarification. Cubic projection is a method of environment mapping that utilizes the six faces of a cube in a 3D Cartesian coordinate system. The environment is projected onto the sides of a cube and stored as six squares. The cube map is generated by first rendering the scene of a position six times, each from a viewpoint, with the views defined by a 90 degree angle of view frustum representing each cube face shown in Figure 1.

Six 90° view frustum square images were captured within *Blender* with a virtual camera at each defined position to map a cubic projection. The defined positions store the latitude, longitude, and altitude. Equation (1) denotes the generation process:

(1) $$p=\left[lat,lon,alt\right]\;Img_{cubic,\;p}^{3DM\text{\_}seg}=C\text{\_}P\left(3DM\text{\_}seg,p\right)$$

where p is the three-dimensional position, 3DM_seg is the segmented building model, and C_P is the function to capture the six images. The cubic projection at a defined position is denoted as $Img_{cubic,\;p}^{3DM\text{\_}seg}$.

### 3.3. Equirectangular Projection Generation

To meet the real-time and low power consumption demands in pedestrian positioning, the BIM pre-computed images and smartphone images are compared in the 2D equirectangular projection frame. This is because equirectangular projection allows a full spherical view of its surroundings, as shown in Figure 1. Hence, at each position, only one equirectangular image is stored.

Equation (2) shows the transformation from the cubic projection into the equirectangular projection at a given position, which requires the conversion from Cartesian coordinates to spherical coordinates:

(2) $$Img_{ERP,\;p}^{3DM\text{\_}seg}=ER\text{\_}P\left(Img_{cubic,\;p}^{3DM\text{\_}seg}\right)$$

where ER_P is the function to convert the cubic projection into the equirectangular projection described in [28]. The equirectangular projection at a defined position is denoted as $Img_{ERP,\;p}^{3DM\text{\_}seg}$.

As for the cubic projection, the defined equirectangular projection positions store the latitude, longitude, and altitude. The format of the generated segmented equirectangular images can be described as:

(3) $$Img_{ERP,\;p}^{3DM\text{\_}seg}=SI\left(\psi_{p},\theta_{p}\right)\;SI\in \left\{\begin{array}{c} \mathrm{Sky}\;\left(0\right),\;\mathrm{Concrete}\;\left(1\right),\;\mathrm{Glass}\;\left(2\right),\;\\ \mathrm{Metal}\;\left(3\right),\;\mathrm{Foliage}\;\left(4\right),\;\mathrm{Others}\;\left(5\right) \end{array}\right\}$$

where $\psi_{p},\theta_{p}$ are the 2D pixel coordinates of the pixel inside the image generated based on the position p. Because the image is equirectangular, each set of pixel coordinates is denoted in rotational elements because it also corresponds to the yaw and pitch. SI is the function that assigns each pixel an indexed number to represent a material class. Each image stores its corresponding position. Figure 1 shows an example of an equirectangular image based on a defined position. The generated images are pre-computed and stored in the smartphone as indexed images to reduce storage size, and used in the online phase for image matching.

### 3.4. Smartphone Image Acquistion and Format

Because the smartphone image is analyzed according to the urban scene, the comparison is likely to perform well when there is a richer and more diverse urban scene. Therefore, the widest available angle lens is the preferred choice because it is more suitable to capture greater information of the surrounding urban scene in the image. A conventional smartphone camera with a 120° diagonal field of view, 4:3 aspect ratio, resolution of [1000,750] pixels was used to capture the images shown in Figure 1.

The smartphone image is first segmented as described in Section 3.8. Then, to match with the candidate images in the equirectangular projection frame, the smartphone image is transformed to the equirectangular projection based on the smartphone intrinsic parameters and the IMU sensor measurement. The intrinsic parameters can be identified in the image EXIF metadata and a lookup database of the smartphone camera sensors.

(4) $$r=\left[\psi ,\theta ,\varphi \right]\;Img_{ERP,r}^{cam\text{\_}seg}=ER\text{\_}P\left(Img^{cam\text{\_}seg},r\right)$$

where r is the three-dimensional rotation estimated by the IMU sensor. The format of the smartphone segmented equirectangular images can be described as:

(5) $$Img_{ERP,r}^{cam\text{\_}seg}=SI\left(\psi ,\theta \right)\;SI\in \left\{\begin{array}{c} \mathrm{Sky}\;\left(0\right),\;\mathrm{Concrete}\;\left(1\right),\;\mathrm{Glass}\;\left(2\right),\;\\ \mathrm{Metal}\;\left(3\right),\;\mathrm{Foliage}\;\left(4\right),\;\mathrm{Others}\;\left(5\right) \end{array}\right\}$$

where  ψ,θ are the 2D pixel coordinates of the pixel inside the image.

As shown in Figure 1, only the transformed area in the smartphone equirectangular image is used to compare against the candidate images; the “black” area is ignored. Images captured at the same position in different angles are therefore be transformed at their respective area in the equirectangular image.

### 3.5. Candidate Position Distribution

Candidate positions are distributed around the initial estimated position. The initial rough estimation of the position is calculated by the smartphone GNSS receiver and IMU when capturing an image with the smartphone. The candidate latitudes and longitudes are distributed around the initial position in a 40 m radius with 1 m resolution. The candidate altitude remains the same as that measured by the smartphone due to its already high accuracy. The candidate rotation is distributed around the initial rotation with 30° yaw, 3° pitch, and 3° roll, with 1° separation. The following distribution values are calibrated by finding the maximum possible error when comparing the smartphone estimated rotation with their ground truth. The positions are then reduced to the specific candidate poses shown in (6):

(6) $$x=\left\{p,r\right\}\;X=\left\{x_{0}\cdots x_{s}\right\}$$

where x is the state (position) containing the 3D position and 3D rotation. s is the index of the positions outside of the buildings, which is generated offline and saved in a database. Candidate position $x_{j}$ is extracted from the database X, where $x_{j}\in \;X$**,** and the subscript *j* is the index of the candidate positions. The corresponding image for each candidate position is denoted as $Img_{ERP,\;p_{j}}^{3DM\text{\_}seg}$. The distributed candidate equirectangular images are then used to compare against the smartphone equirectangular images, $Img_{ERP,\;r_{j}}^{cam\text{\_}seg}$.

### 3.6. Hand Labelled Material Segmentation

The captured smartphone images were labelled manually with the Image Labeler application in MATLAB. In the future, however, we plan to utilize a deep learning neural network to automatically identify the material. This is discussed in further detail in Section 5. The smartphone image is then hand labelled to output the ideally segmented smartphone image.

(7) $$Img^{cam\text{\_}seg}=H\text{\_}L\left(Img^{cam\text{\_}raw}\right)$$

where H_L is the function to manually segment the smartphone image.

### 3.7. Material Matching

In the online stage, the candidate images are compared to the smartphone image. The matching algorithm calculates the score of each candidate image. The target function aims to identify the candidate image with the largest similarity with respect to the semantic information of the materials. A typical approach is to use the region and contours of each material class in the candidate image to compare with the corresponding material class in the smartphone image. Because the candidate images generated from the BIM do not have foliage and dynamic objects, any “foliage” and “other” classes identified in the smartphone image are excluded from the similarity calculation.

#### 3.7.1. Dice Metric

We used the Sørensen–Dice coefficient metric to compare the region of two material segmented images [29]. Equation (8) shows the calculation of the similarity index for each material class:

(8) $$sim_{class}^{di}\left(Img_{ERP,\;r_{j}}^{cam\text{\_}seg},Img_{ERP,\;p_{j}}^{3DM\text{\_}seg}\right)=\frac{\left|Img_{ERP,\;p_{j}}^{3DM\text{\_}seg}\left(class\right)\cap^{\;}Img_{ERP,\;r_{j}}^{cam\text{\_}seg}\left(class\right)\right|}{0.5\left(N_{class,ERP,\;p_{j}}^{3DM\text{\_}seg}+N_{class,ERP,\;r_{j}}^{cam\text{\_}seg}\right)}$$

where class is the index that represents a material, and $sim_{class}^{di}\left(Img_{ERP,\;r_{j}}^{cam\text{\_}seg},Img_{ERP,\;p_{j}}^{3DM\text{\_}seg}\right)$ is the similarity index of the smartphone image and the candidate image for a material class. A measure to consider is the ratio of the detected region compared to the total image size. A smaller matched region should have lower weighting, whereas a larger matched region should have higher weighting. Therefore, the similarity of each segmented material needs to be weighted according to the number of pixels it occupies in the candidate image to calculate the score of each class, represented in (9):

(9) $$N_{class,ERP,\;p_{j}}^{3DM\text{\_}seg}=\left|Img_{ERP,\;p_{j}}^{3DM\text{\_}seg}\left(class\right)\right|\;score_{class}^{di}\left(x_{j}\right)=sim_{class}^{di}\left(Img_{ERP,\;r_{j}}^{cam\text{\_}seg},Img_{ERP,\;p_{j}}^{3DM\text{\_}seg}\right)\cdot \left(N_{class,ERP,\;p_{j}}^{3DM\text{\_}seg}/N_{\mathrm{total}}\right)$$

where $N_{class,ERP,\;p_{j}}^{3DM\text{\_}seg}$ is the pixel region of a material class in the candidate image, and $N_{\mathrm{total}}$ is the total number of class pixels in the image. The dice score of a class is denoted as $score_{class}^{di}\left(x_{j}\right)$. Finally, the score for each material is combined to obtain the score of the candidate, as shown in (10):

(10) $$score^{di}\left(x_{j}\right)=\underset{class}{\sum }score_{class}^{di}\left(x_{j}\right)$$

#### 3.7.2. Jaccard Metric

The Jaccard coefficient metric is similar to the Dice coefficient metric, but instead satisfies the triangle inequality and measures the intersection over the union of the labelled region [30]. We also used the Jaccard coefficient metric to compare the region of two material segmented images. Equation (11) demonstrates the calculation of the similarity index for each material class:

(11) $$sim_{class}^{ja}\left(Img_{ERP,\;r_{j}}^{cam\text{\_}seg},Img_{ERP,\;p_{j}}^{3DM\text{\_}seg}\right)=\frac{\left|Img_{ERP,\;p_{j}}^{3DM\text{\_}seg}\left(class\right)\cap^{\;}Img_{ERP,\;r_{j}}^{cam\text{\_}seg}\left(class\right)\right|}{\left|Img_{ERP,\;p_{j}}^{3DM\text{\_}seg}\left(class\right)\cup^{\;}Img_{ERP,\;r_{j}}^{cam\text{\_}seg}\left(class\right)\right|}$$

where $sim_{class}^{ja}\left(Img_{ERP,\;r_{j}}^{cam\text{\_}seg},Img_{ERP,\;p_{j}}^{3DM\text{\_}seg}\right)$ is the similarity index of the smartphone image and the candidate image for a material class. As for the former metric, the similarity for each segmented material needs to be weighted according to the number of pixels it occupies in the candidate image to calculate the score of each class, as represented in (12):

(12) $$score_{class}^{ja}\left(x_{j}\right)=sim_{class}^{ja}\left(Img_{ERP,\;r_{j}}^{cam\text{\_}seg},Img_{ERP,\;p_{j}}^{3DM\text{\_}seg}\right)\cdot \left(N_{class,ERP,\;p_{j}}^{3DM\text{\_}seg}/N_{\mathrm{total}}\right)$$

The score of a class is denoted as $score_{class}^{ja}\left(x_{j}\right)$. Finally, the score for each material is combined to obtain the score for each candidate shown in (13).

(13) $$score^{ja}\left(x_{j}\right)=\underset{class}{\sum }score_{class}^{ja}\left(x_{j}\right)$$

#### 3.7.3. Boundary F1 Metric

The contour quality significantly contributes to the perceived segmentation quality. The benefit of the Boundary F1 (BF) metric is that it evaluates the accuracy of the segmentation boundaries [31], which are not captured by the Dice and Jaccard metrics because they are regional-based metrics.

Let us call $B_{ERP,\;r_{j}}^{cam\text{\_}seg}\left(class\right)$ the boundary of the class of $Img_{ERP,\;r_{j}}^{cam\text{\_}seg}\left(class\right)$, and similarly $B_{ERP,\;p_{j}}^{3DM\text{\_}seg}\left(class\right)$ the boundary of the class of $Img_{ERP,\;p_{j}}^{3DM\text{\_}seg}$. For a distance threshold of 5 pixels, the metric disregards the content of the segmentation beyond the threshold distance of 5 pixels under which boundaries are matched. The precision for a class is defined as:

(14) $$P_{class}\left(x_{j}\right)=\frac{1}{\left|B_{ERP,\;p_{j}}^{3DM\text{\_}seg}\right|}\underset{b\in B_{ERP,\;p_{j}}^{3DM\text{\_}seg}\left(class\right)}{\sum }⟦d\left(b,B_{ERP,\;r_{j}}^{cam\text{\_}seg}\left(class\right)\right)<5⟧$$

The recall for a class is defined as:

(15) $$R_{class}\left(x_{j}\right)=\frac{1}{\left|B_{ERP,\;r_{j}}^{cam\text{\_}seg}\right|}\underset{b\in B_{ERP,\;r_{j}}^{cam\text{\_}seg}\left(class\right)}{\sum }⟦d\left(b,B_{ERP,\;p_{j}}^{3DM\text{\_}seg}\left(class\right)\right)<5⟧$$

where ⟦⟧ represents the Iverson bracket notation, and ⟦s⟧=1 if ⟦s⟧=true and 0 otherwise, and d() denotes the Euclidean distance measured in pixels. The Boundary F1 measure for a class is given by:

(16) $$score_{class}^{bf}\left(x_{j}\right)=\frac{2\cdot P_{class}\left(x_{j}\right)\cdot R_{class}\left(x_{j}\right)}{R_{class}\left(x_{j}\right)+P_{class}\left(x_{j}\right)}$$

The BF score of a class is denoted as $score_{class}^{bf}\left(x_{j}\right)$. Finally, the score for each material is combined by averaging the score over all classes present in the candidate image to obtain the total score for each candidate, as shown in (17):

(17) $$score^{bf}\left(x_{j}\right)=\frac{1}{n\text{\_}class}\underset{class}{\sum }score_{class}^{bf}\left(x_{j}\right)$$

where n_class is the total number of classes; in this research, we used six classes.

### 3.8. Combined Material Matching

We considered the score of each method (Dice, Jaccard, BF) for the 9 tested images described in Section 4 to calibrate their respective CDF based on a Gaussian distribution. The scores of each method are used to calculate the corresponding probability value in their respective distributions as shown in Table 1:

(18) $$prob^{*}\left(x_{j}\right)=\frac{1}{\sigma^{*}\cdot \sqrt{2\pi }}\cdot \overset{score^{*}\left(x_{j}\right)}{\underset{-\infty }{\int }}e^{-\frac{1}{2}{\left(\frac{x-\mu^{*}}{\sigma^{*}}\right)}^{2}}dx$$

where ∗ is the variable that is dependent on the method, σ is the standard deviation, and μ is the mean of the CDF.

The combined probability becomes the likelihood of each candidate:

(19) $$likelihood\left(x_{j}\right)=prob^{di}\left(x_{j}\right)\cdot prob^{ja}\left(x_{j}\right)\cdot prob^{bf}\left(x_{j}\right)$$

### 3.9. Position Solution

A higher priority is given to the candidate image with a higher likelihood. In theory, the candidate image at the ground truth should have the maximum likelihood. Thus, the candidate with the maximum likelihood is selected as the chosen candidate, as indicated in (20):

(20) $$\hat{x}=\arg\underset{x_{j}}{\max}\left(likelihood\left(x_{j}\right)\right)$$

where $\arg\;\underset{x_{j}}{\max}$ is a function that filters the highest total score, and $\hat{x}$ is the estimated candidate pose with the highest likelihood. The chosen candidate position stores the latitude, longitude, altitude, yaw, pitch, and roll.

## 4. Experimental Results

### 4.1. Image and Test Location Setting

In this study, the experimental locations were selected within the Tsim Sha Tsui and Hung Hom areas of Hong Kong, as shown in Table 2. Three locations were selected in challenging deep urban canyons surrounded by tall buildings where GNSS signals are heavily reflected and blocked. Three images were taken at each of the selected locations using a generic smartphone camera (Samsung Galaxy Note 20 Ultra 5G smartphone with an ultra-wide 13mm 12-MP f/2.2 lens) and a tripod. The experimental ground truth positions were determined based on Google Earth and nearby identifiable landmarks, such as a labelled corner on the ground. Based on the experience of previous research [18,32], the ground truth uncertainty of latitude and longitude was ±1m and yaw was ±2°. The pitch and roll angles were measured using the *XPRO geared head, Manfrotto*, with ±1° uncertainty.

The experimental images were chosen with the following skyline categorizations: distinctive, symmetrical, insufficient, obscured, and concealed. Categorizations were based on the difficulties experienced by current 3DMA GNSS and vision-based positioning methods. The smartphone was used to capture the images and to record the low-cost GNSS position and IMU rotation. The GNSS receiver within the smartphone was a Broadcom BCM47755. The IMU was a LSM6DSO MEMS and was designed by STMicroelectronics. Images were taken at each location with different combinations of scenic features to demonstrate the proposed semantic VPS method. The locations were chosen to test the following environments: dense foliage (Loc. 1), street (Loc. 2), and alleyway (Loc. 3).

### 4.2. Positioning Results Using Ideal Segmentation

The positioning quality of the proposed method was analyzed based on the ideal manual segmentation of the smartphone image. The experimental results were then post-processed and compared to the ground truth and different positioning algorithms as shown in Table 3, including:

1. Proposed semantic VPS (Combination of Dice, Jaccard and BF Metrics)
2. Proposed semantic VPS (Dice only)
3. Proposed semantic VPS (Jaccard only)
4. Proposed semantic VPS (BF only)
5. Skyline Matching: Matching using sky and building class only [21].
6. 3DMA: Integrated solution by 3DMA GNSS algorithm on shadow matching, skymask 3DMA and likelihood based ranging GNSS [33].
7. WLS: Weighted Least Squares [34].
8. NMEA: Low-cost GNSS solution by Galaxy S20 Ultra, Broadcom BCM47755.

Loc. 1 is in an urban environment with dense foliage, which contains multiple non-distinctive medium-rise buildings. The results show the positioning accuracy of the proposed semantic VPS improves upon the existing advanced positioning methods. An error of approximately 5.56 m from the smartphone ground truth suggests that the semantic VPS can be used as a positioning method in foliage dense environments. Utilizing additional material information from buildings, this approach increases the performance of skyline matching by three-fold. The inability of skyline matching was due to the presence of foliage obscuring the skyline. Without an exposed skyline, a correct match cannot be obtained and the positioning error may be increased. 3DMA was shown to correct the positioning to a higher degree, ranking behind the proposed method. The positioning errors of WLS and NMEA were likely because of the diffraction of the GNSS signals passing under the foliage with the combination of high-rise buildings.

As shown in the heatmap in Table 4, the proposed method using the Dice and Jaccard metrics have very large positioning errors, possibly due to the lack of distinctive materials captured in the smartphone image. The tested location is surrounded by buildings of the same shape, size, and material. Therefore, it is a very challenging environment for the proposed method because the candidate images share a common material distribution. It can be seen in this situation that using the BF achieves a higher positioning accuracy than the Dice and Jaccard metrics, because it calculates the material contour rather than the material region. Thus, with the combination of the three metrics, this foliage dense environment proved suitable for the proposed method, which successfully utilized materials as information for matching.

Loc. 2 is in a common street urban environment with high-rise buildings. The results show that the positioning accuracy of the proposed method improves the positioning accuracy to around two meters. In an environment where skyline matching should perform the best, the proposed method also improves skyline matching by more than three-fold. The matching of the diverse materials distributed in the scene, in addition to the distinctive skyline, significantly improved the positioning accuracy. 3DMA lagged slightly behind skyline matching, whereas WLS increased the positioning error. It should be noted that the estimated positioning error for the NMEA is around 8 m, which is significantly less than that of Loc. 1. This is likely due to the relative open area along the street, as shown in Table 2.

The heatmap results shown in Table 4 demonstrate that the metrics complement each other when combined. As shown in Loc. 2.1, in a scene with diverse materials, the Dice and Jaccard metrics have a higher positioning accuracy and achieve a higher likelihood than BF. Therefore, the combination of the three metrics supports regional-based similarities.

Loc. 3 is clearly the most challenging urban environment for the 3DMA GNSS and vision-based positioning methods due to the close and compact high-rise buildings and visually symmetrical features. It can be seen that all methods suffer in this environment, and most noticeably WLS. The results show that the positioning error of the proposed method is nearly 16 m and can be improved significantly. Nonetheless, it should be noted that this is a 35% improvement in positioning compared to skyline matching. Due to the lack of a distinctive skyline, skyline matching can potentially increase the positioning error if matched with the wrong image, as demonstrated at this position. 3DMA lags behind the proposed method and, as demonstrated, only the proposed method and 3DMA slightly improved the positioning accuracy.

The poor results can be explained by two conditions required for accurate positioning. Firstly, the images ideally should have no segmentation error. This error is not considered in the positioning results, because we are assessing the ideal image segmentation. Instead, we analyzed the segmentation error in relation to the positioning error in Section 4.4. Secondly, ideally there should be no discrepancies between the smartphone image and the candidate image at ground truth. Loc. 3 suffers from the latter as shown in Table 5.

This error is shown in the positioning results of Loc. 3, where many candidates share a common similarity and color. Thus, it is important to ensure the BIM is constantly updated to reflect reality.

### 4.3. Rotational Results Using Ideal Segmenatation

The three-dimensional rotational performance of the proposed method was analyzed based on the ideal smartphone image segmentation, then compared to the smartphone IMU as shown in Table 6.

The results show that, in an urban environment with features, the materials of buildings can be used to estimate the rotation. The yaw, pitch, and roll have an accuracy of 2.3, 1.4 and 1.3 degrees, respectively. However, the smartphone IMU pitch and roll estimation is already very accurate compared to the proposed method, and thus the proposed method only degrades the estimation. Instead, the proposed method succeeds at predicting the yaw accurately, within an average of 2.3 degrees. Hence, the proposed method can be considered an accurate approach to estimate the heading of the user in an urban environment.

Therefore, it is suggested that the proposed method should use the already accurate altitude, pitch, and roll for position, and the yaw estimation. Eliminating the estimation of three dimensions will significantly reduce computational load because fewer candidate images are used for matching.

### 4.4. Segmentation Accuracy vs. Localization Results

To test the effect of the semantic segmentation accuracy on the localization results, we considered the two conditions required for accurate positioning. Ideally, there should be no segmentation error and no discrepancies between the smartphone image and the candidate image at the ground truth. We can therefore further classify these two types of errors: contour-based error and regional-based error. In our experiments, we tested whether discrepancies can contribute heavily to the positioning accuracy, as shown in Table 4, where the smartphone image differs from the candidate image at the ground truth. Therefore, we can consider this as a regional-based error because the entire region differs between the images. We should also consider the contour-based error, which is not demonstrated in our experiments, but is reflected in a realistic output of a semantic segmentation neural network where the boundaries of a region are shifted. Contour error can be problematic for boundary related metrics, such as the BF metric, which focus on the evaluation along the object edges. Correctly identifying these edges is very important, because any shift in alignment can lead to a mismatch with another candidate image. Thus, we considered the candidate images at the ground truth to be the ideal images, because there are no regional-based or contour-based errors. We purposely mislabeled the ideal images by adding the two types of noise to model the amount of segmentation accuracy.

**Figure 2:**
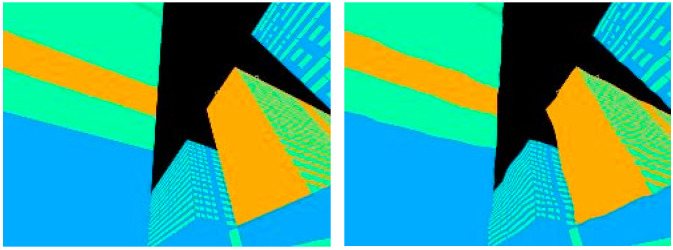
Example of a candidate image on the left, and a slightly elastically distorted candidate image on the right.

**Figure 3:**
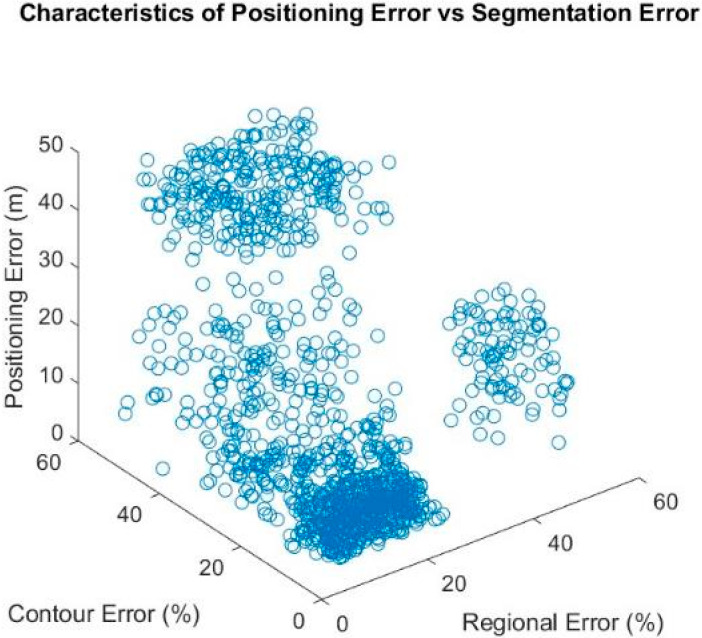
The effects of contour-based error and regional-based error on the positioning error of the proposed semantic-based VPS.

To model the two types of errors, we performed a Monte Carlo simulation. We elastically distorted the ideal image randomly to generate over 1000 distorted images described in [35], each with a distinctive regional-based and contour-based error. We then compared the distorted image with the ideal image using two metrics, the combined Dice and Jaccard metric for regional-based error, and the BF metric for the contour-based error. We then used our proposed method to obtain a positioning error by comparing the positioning solution of the distorted image with the ground truth position. Figure 2 shows the candidate image with the contour mislabeled using the elastic distortion algorithm. Figure 3 shows the characteristics of position error in the presence of segmentation error.

The results show a good positioning accuracy at lower levels of segmentation error. It can be seen the positioning error in the 0 to 20% segmentation error range is approximately 0–5 m. However, the proposed method begins to suffer when incorrect segmentation reaches more than 20% for contour-based errors and 25% for regional-based errors. This is followed by a deteriorating positioning performance, where the positioning error increases to 10–20 m. At 40% contour- and regional-based errors, the matching algorithm fails to perform accurately, increasing the risk of greater positioning error. It can be seen at this segmentation error range, the distorted image matches with random incorrect candidate images; thus, the positioning error spreads across a wide region.

The Monte Carlo simulation results demonstrate the importance of a correct contour-based and regional-based segmentation and suggests that, to successfully utilize the proposed method with a high positioning accuracy, a semantic segmentation neural network with no less than 80% segmentation accuracy is preferred. The results also suggest disabling the proposed method when the smartphone image is matched with a candidate image with a segmentation difference of more than 20–25%. In such situations, relying on other advanced positioning techniques such as 3DMA would likely yield better positioning results.

### 4.5. Discussion on Validity and Limitation

The proposed method presented in this research permits self-localization based on material that is widely distributed among urban scenes. Provided that the smartphone image segmentation is ideal, experiments show that our approach outperforms the positioning performance of the current state-of-the-art methods by 45% and improves the yaw performance by eight-fold compared to smartphone IMU sensors.

The pitch and roll estimated by the proposed method, however, achieves a lower performance by half a degree compared to the smartphone IMU sensors. Hence, it is suggested that the proposed method uses the already accurate pitch and roll estimated by the smartphone IMU sensors. The elimination of altitude, pitch, and yaw estimation will significantly reduce computational load because fewer images are used for matching.

Another limitation is due to inaccurate segmentation. As demonstrated in this research, the BIM was out of date, leading to discrepancies between the smartphone image and images at the ground truth. It was shown that when the segmentation error is greater than 20–25%, the positioning performance deteriorates significantly. Therefore, it is necessary to frequently update the utilized 3D city model.

## 5. Conclusion and Future Work

### 5.1. Conclusions

This paper proposes a semantic VPS solution for position (six-DOF) estimation by introducing materials as a source of information. In short, the semantic information of materials is extracted from the smartphone image and compared to the BIM generated images. Multiple image matching metrics were tested to accurately identify the position of the generated image that is closest to the smartphone image.

Existing 3DMA vision-integrated approaches for urban positioning use either edge features or skylines for positioning. This study proposed a method that extends these paradigms to formulate the positioning as a semantic-based problem using material as the semantic information. Our experiments demonstrate that it is possible to outperform existing GNSS and advanced GNSS positioning methods in urban canyons. The advantages of the semantic VPS method are numerous:

- The formulation of positioning as a semantic-based problem enables us to apply the existing wide variety of advanced optimization/shape matching metrics to the problem.
- Materials are diverse, distinctive, and widely distributed; hence, the semantic information in an image can be easily recognized.
- The utilization of building materials for positioning eliminates the need for skyline and building boundary reliance.
- Foliage and dynamic objects are considered for positioning.
- The semantics of buildings stored as vector maps can be simply and accurately updated and labeled.

Based on the results presented in this paper, we conclude the proposed method improves on the latitude, longitude, and heading estimation of existing advanced positioning methods.

### 5.2. Future Work

Several potential future developments are suggested.

- Research has shown it is possible to identify a wide variety of materials in images in the indoor environment [36]. Therefore, it is suggested to develop and train a deep learning neural network to identify materials in smartphone images in the outdoor environment for real-time use. Improvement in the deep learning neural network may also aid automatic segmentation of 3D building models, reducing the offline preparation time.
- By adding the common building material classes and dynamic objects to aid differentiation (including concrete, stone, glass, metal, wood, bricks, pedestrians, cars, etc.), given a large and high-quality dataset, the proposed method can be adapted to a variety of different uses.
- It is possible to provide computation of depth based on the BIM and the virtual camera, which can then be stored as additional information in the generated images. This depth information can allow precise AR after image matching.
- To maximize all available visual information, the semantic VPS can also make use of objects in addition to materials, or the combination of a semantic VPS and a feature-based VPS, to yield better positioning performance.
- To reduce storage and computational load, the images can be stored as contour coordinates rather than pixels.
- The semantic VPS may also be further improved by extending the functionality to work in different weather, time, and brightness conditions.
- One difficulty encountered in this experiment was the discrepancy between reality and the BIM; hence, it is suggested to use a crowdsourcing map to continuously update the model.
- For dynamic positioning, a multiresolution framework can be used, where the search starts from a big and sparse grid and is then successively refined on smaller and denser grids. Thus, the position of the chosen candidate is used to refine a smaller search area.

The average time taken to estimate a single point position in a 40 m radius is 10 s, which can be reduced to within 2 s by refining to a smaller search area (5 m) during dynamic positioning.

## Figures and Tables

**Table 1:** Parameters of the Gaussian distribution.

Method	Standard Deviation (σ)	Mean (μ)
Dice	0.1813	0.6686
Jaccard	0.1567	0.5399
BF	0.1387	0.4275

**Table 2:** Locations and images tested with the proposed semantic VPS method.

Loc.	Experimental Images			
1	The Hong Kong Polytechnic University, Hung Hom			
	Overview	1.1	1.2	1.3
	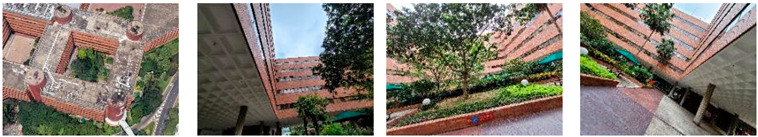			
	Overview	Obscured	Concealed	Obscured
2	Isquare, Tsim Sha Tsui			
	Overview	2.1	2.2	2.3
	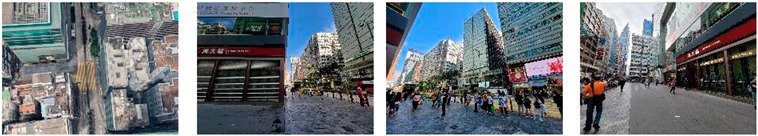			
	Overview	Distinctive	Distinctive	Distinctive
3	East Tsim Sha Tsui			
	Overview	3.1	3.2	3.3
	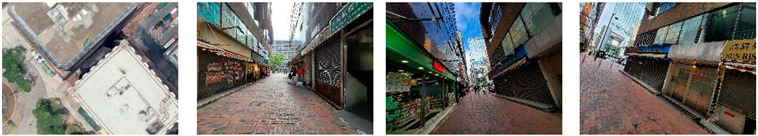			
	Overview	Symmetrical	Insufficient	Insufficient

**Table 3:** Positioning performance comparison of the proposed semantic VPS and other advanced positioning algorithms.

Loc.	Deviation from Ground Truth Error. Unit: Meter.				
	Semantic VPS (Combined)	Skyline Matching	3DMA	WLS	NMEA
1.1	7.07	22.92	7.96	17.66	36.24
1.2	4.34	22.62			
1.3	5.28	7.14			
1. Avg.	5.56	17.56			
2.1	0.66	14.80	6.87	23.29	7.94
2.2	1.83	1.58			
2.3	3.43	2.89			
2. Avg.	1.97	6.42			
3.1	29.89	13.57	18.80	46.58	18.89
3.2	6.61	25.53			
3.3	10.53	24.80			
3. Avg.	15.68	21.30			
All Avg.	7.74	15.09	11.21	29.18	21.02

**Table 4:** Heatmap of the likelihood of candidate images compared to the smartphone image based on the proposed semantic VPS method.

Loc.	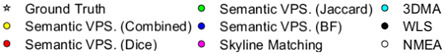			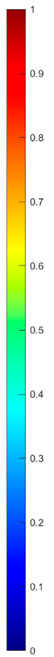
1	1.1	1.2	1.3	
	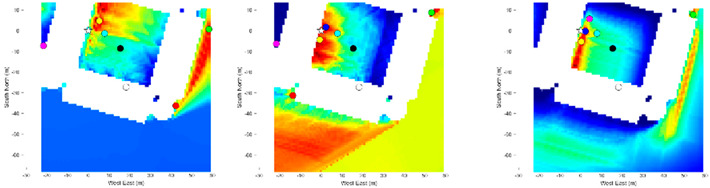			
2	2.1	2.2	2.3	
	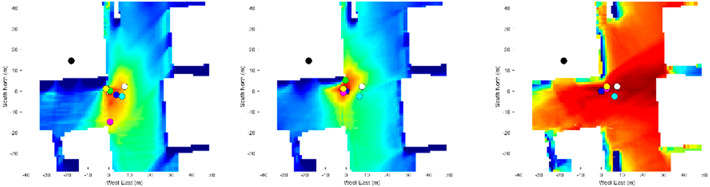			
3	3.1	3.2	3.3	
	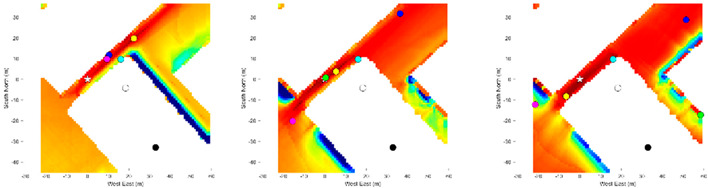			

**Table 5:** Discrepancy between reality and BIM.

	Reality	BIM
Textured	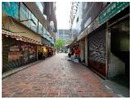	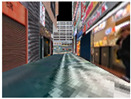
Labelled	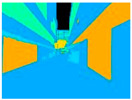	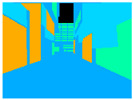

**Table 6:** Heatmap of the likelihood of candidate images compared to the smartphone image based on the proposed semantic VPS method.

Loc.	Deviation from Ground Truth. Unit: Degrees.					
	Semantic VPS			Smartphone IMU		
	ψ	θ	φ	ψ	θ	φ
1.1	−4	0	−1	−27	−2.0	1.0
1.2	3	2	−2	7	0.5	−0.5
1.3	3	2	-1	18	−0.5	0.5
1. Avg.	3.3	1.3	1.3	17.3	1.0	0.6
2.1	5	1	−2	11	0.5	−1.0
2.2	−3	−1	0	18	2.0	0.0
2.3	1	2	−2	19	−2.0	0.5
2. Avg.	3	1.3	1.3	16	1.5	0.5
3.1	2	2	−2	31	1.0	−1.5
3.2	0	1	0	28	0.5	−0.2
3.3	0	−2	−2	27	−0.5	−0.2
3. Avg.	0.6	1.7	1.3	28.6	0.6	1.8
All Avg.	2.3	1.4	1.3	20.6	1.0	1.0
